# Supplementary material for: High-pH structure of EmrE reveals the mechanism of proton-coupled substrate transport
Source: Nat Commun. 2022 Feb 18;13:991. doi: 10.1038/s41467-022-28556-6 (PMC8857205; doi:10.1038/s41467-022-28556-6)
Supplement: Supplementary file 1 — Supplementary Information [file 41467_2022_28556_MOESM1_ESM.pdf]

Supplementary Information

High-pH Structure of EmrE Reveals the Mechanism of Proton-Coupled Substrate Transport

Alexander A. Shcherbakov [1], Peyton J. Spreacker [2], Aurelio J. Dregni [1], Katherine A. Henzler-Wildman [2], and Mei Hong [1]*

[1] Department of Chemistry, Massachusetts Institute of Technology, 170 Albany Street, Cambridge, MA 02139

[2] Department of Biochemistry, University of Wisconsin at Madison, Madison, WI 53706

This PDF file includes:

Tables S1-S6
Supplementary Figures 1 to 11

Supplementary Table 1. Explanation of the eight ^{1}H-detected 3D correlation MAS NMR experiments for assigning the chemical shifts of high-pH EmrE in DMPC bilayers. OaB = Out-and-Back. All H-N and H-C transfers used dipolar cross polarization.

Experiment	F1-F2-F3	Connectivity	Polarization transfer
hCANH	$CA_i – N_i – H^N_i$	Intra-residue	Dipolar transfer for CA-N
hcaCBcaNH	$CB_i – N_i – H^N_i$	Intra-residue	OaB INEPT for CB-CA, dipolar CA-N
hCOcaNH	$CO_i – N_i – H^N_i$	Intra-residue	Dipolar CO-CA, CA-N
hCAcoNH	$CA_{i-1} – N_i – H^N_i$	Inter-residue	Dipolar CA-CO and CO-N
hCONH	$CO_{i-1} – N_i – H^N_i$	Inter-residue	Dipolar CO-N
hcaCBcacoNH	$CB_{i-1} – N_i – H^N_i$	Inter-residue	OaB INEPT for CB-CA, dipolar CA-CO, CO-N
hNcacoNH	$N_{i-1} – N_i – H^N_i$	Inter-residue	Dipolar N-CA, CA-CO, CO-N
HncacoNH	$H^N_{i-1} – N_i – H^N_i$	Inter-residue	Dipolar N-CA, CA-CO, CO-N

Supplementary Table 2. Detailed H^N-F distance constraints between EmrE and F_4-TPP^+ used for HADDOCK docking of the ligand into the protein and for structure-based assignment of the H^N-^{19}F pairs.

Residue	Atom	r_{HF} (Å)	δ_{low} (Å)	δ_{high} (Å)	Assigned ^{19}F Atoms (Run 2)			
G8A	HN	9.7	2.0	40.0	F3	F13	F18	F8
G9A	HN	12.0	3.8	40.0	F3	F13	F18	F8
A10A	HN	12.0	3.9	40.0	F3	F13	F18	F8
I11A	HN	8.4	3.1	40.0	F3	F13	F18	F8
A13A	HN	8.7	1.3	40.0	F3	F13	F18	F8
E14A	HN	7.8	2.8	1.8	F8			
V15A	HN	6.3	2.5	0.5	F8			
I16A	HN	9.3	1.6	40.0	F3	F13	F18	F8
G17A	HN	7.2	2.7	1.1	F8			
T18A	HN	7.7	0.8	2.2	F8			
T19A	HN	8.7	2.3	40.0	F3	F13	F18	F8
L20A	HN	9.2	2.6	40.0	F3	F13	F18	F8
M21A	HN	9.6	2.9	40.0	F3	F13	F18	F8
K22A	HN	12.0	3.8	40.0	F3	F13	F18	F8
F23A	HN	12.0	3.8	40.0	F3	F13	F18	F8
S24A	HN	10.7	3.8	40.0	F3	F13	F18	F8
E25A	HN	12.0	3.8	40.0	F3	F13	F18	F8
G26A	HN	12.0	3.8	40.0	F3	F13	F18	F8
F27A	HN	12.0	3.8	40.0	F3	F13	F18	F8
R29A	HN	11.5	3.4	40.0	F3	F13	F18	F8
L30A	HN	11.9	3.9	40.0	F3	F13	F18	F8
W31A	HN	11.3	3.3	40.0	F3	F13	F18	F8
S33A	HN	10.3	2.5	40.0	F3	F13	F18	F8
V34A	HN	10.6	2.6	40.0	F3	F13	F18	F8
G35A	HN	12.0	4.9	40.0	F3	F13	F18	F8
C39A	HN	8.9	3.4	40.0	F3	F13	F18	F8
Y40A	HN	7.3	2.6	1.1	F8			
C41A	HN	12.0	5.9	40.0	F3	F13	F18	F8
S43A	HN	4.3	2.3	1.3	F8			
F44A	HN	4.8	1.8	0.8	F8			
Q49A	HN	10.8	3.9	40.0	F3	F13	F18	F8
G57A	HN	8.1	3.0	40.0	F3	F13	F18	F8
I58A	HN	11.7	3.6	40.0	F3	F13	F18	F8
A59A	HN	9.5	3.8	40.0	F3	F13	F18	F8
Y60A	HN	6.3	2.7	0.5	F3			
I62A	HN	8.2	3.1	40.0	F3	F13	F18	F8
W63A	HN	8.2	3.1	40.0	F3	F13	F18	F8
V64A	HN	6.2	2.7	0.5	F3			
G65A	HN	6.7	0.6	0.7	F3			
V66A	HN	10.3	4.4	40.0	F3	F13	F18	F8
G67A	HN	5.9	0.7	0.7	F3			
I68A	HN	7.2	2.6	0.9	F3			
V69A	HN	9.7	4.0	40.0	F3	F13	F18	F8
I71A	HN	8.8	1.3	40.0	F3	F13	F18	F8
S72A	HN	12.0	5.9	40.0	F3	F13	F18	F8
L74A	HN	11.4	3.4	40.0	F3	F13	F18	F8
S75A	HN	7.9	2.9	2.7	F18			
W76A	HN	12.0	4.8	40.0	F3	F13	F18	F8
G77A	HN	9.4	1.7	40.0	F3	F13	F18	F8
F79A	HN	8.7	2.2	40.0	F3	F13	F18	F8
G80A	HN	11.1	3.1	40.0	F3	F13	F18	F8
Q81A	HN	11.8	3.8	40.0	F3	F13	F18	F8

R82A	HN	12.0	3.9	40.0	F3	F13	F18	F8
L83A	HN	11.5	3.5	40.0	F3	F13	F18	F8
D84A	HN	9.5	1.8	40.0	F3	F13	F18	F8
A87A	HN	8.6	1.3	40.0	F3	F13	F18	F8
G90A	HN	11.6	3.6	40.0	F3	F13	F18	F8
C95A	HN	10.9	2.9	40.0	F3	F13	F18	F8
G97A	HN	12.0	3.8	40.0	F3	F13	F18	F8
L103A	HN	8.8	1.4	40.0	F3	F13	F18	F8
L104A	HN	12.0	3.9	40.0	F3	F13	F18	F8
S105A	HN	12.0	3.8	40.0	-			
R106A	HN	10.7	2.7	40.0	-			
Y6B	HN	9.2	1.6	40.0	F3	F13	F18	F8
G8B	HN	10.2	2.4	40.0	F3	F13	F18	F8
G9B	HN	7.3	2.8	1.2	F13			
A10B	HN	8.3	1.1	40.0	F3	F13	F18	F8
I11B	HN	8.5	3.2	40.0	F3	F13	F18	F8
G17B	HN	7.6	2.8	1.8	F13			
T18B	HN	7.4	0.4	0.6	F13			
T19B	HN	11.3	3.2	40.0	F3	F13	F18	F8
K22B	HN	12.0	3.8	40.0	F3	F13	F18	F8
E25B	HN	11.2	3.2	40.0	F3	F13	F18	F8
G26B	HN	10.4	2.5	40.0	F3	F13	F18	F8
R29B	HN	10.1	2.3	40.0	F3	F13	F18	F8
L30B	HN	11.0	3.0	40.0	F3	F13	F18	F8
W31B	HN	9.8	2.1	40.0	F3	F13	F18	F8
S33B	HN	10.1	2.3	40.0	F3	F13	F18	F8
V34B	HN	12.0	3.9	40.0	F3	F13	F18	F8
G35B	HN	12.0	3.8	40.0	F3	F13	F18	F8
S43B	HN	6.9	2.7	0.8	F13			
L47B	HN	8.2	3.0	40.0	F3	F13	F18	F8
Q49B	HN	9.6	3.9	40.0	F3	F13	F18	F8
L51B	HN	9.0	1.5	40.0	F3	F13	F18	F8
A52B	HN	11.2	3.2	40.0	F3	F13	F18	F8
Y53B	HN	9.6	1.9	40.0	F3	F13	F18	F8
G57B	HN	11.9	3.8	40.0	F3	F13	F18	F8
I58B	HN	11.8	3.7	40.0	F3	F13	F18	F8
A59B	HN	9.4	1.8	40.0	F3	F13	F18	F8
Y60B	HN	6.6	2.5	0.6	F18			
A61B	HN	9.9	3.1	40.0	F3	F13	F18	F8
I62B	HN	9.3	3.6	40.0	F3	F13	F18	F8
W63B	HN	9.2	3.7	40.0	F3	F13	F18	F8
V66B	HN	8.5	2.2	40.0	F3	F13	F18	F8
G67B	HN	9.1	1.6	40.0	F3	F13	F18	F8
I68B	HN	9.0	2.5	40.0	F3	F13	F18	F8
V69B	HN	9.8	2.0	40.0	F3	F13	F18	F8
S75B	HN	11.5	3.4	40.0	F3	F13	F18	F8
W76B	HN	9.1	1.5	40.0	F3	F13	F18	F8
G77B	HN	12.0	3.9	40.0	F3	F13	F18	F8
F78B	HN	12.0	3.8	40.0	F3	F13	F18	F8
F79B	HN	12.0	3.8	40.0	F3	F13	F18	F8
G80B	HN	10.6	2.7	40.0	F3	F13	F18	F8
Q81B	HN	10.0	2.2	40.0	F3	F13	F18	F8
R82B	HN	12.0	3.8	40.0	F3	F13	F18	F8
L83B	HN	11.6	3.6	40.0	F3	F13	F18	F8
L85B	HN	12.0	3.9	40.0	F3	F13	F18	F8
A87B	HN	12.0	3.8	40.0	F3	F13	F18	F8

G90B	HN	12.0	3.9	40.0	F3	F13	F18	F8
G97B	HN	12.0	3.8	40.0	F3	F13	F18	F8
L103B	HN	12.0	3.8	40.0	F3	F13	F18	F8
L104B	HN	11.2	3.1	40.0	F3	F13	F18	F8
S105B	HN	10.1	2.3	40.0	-			
R106B	HN	12.0	3.9	40.0	-			
W63B	HE1	3.8	3.8	2.0	F13			

Supplementary Table 3. Distance violation statistics for the 25 lowest-violation structures from HADDOCK structure calculation and detailed violation analysis for two complexes selected for further refinement in MD simulations. The violation detail tables indicate distances from specified protein atoms to each of the four ligand fluorines, to score against the original four-fold ambiguous dataset.

Output Model	Number of Violations	Average Violation (Å)	Std. Dev. Violation (Å)	Sum Total of Violations (Å)
complex_167w	6	0.4	0.2	2.3
complex_189w	6	0.4	0.2	2.3
complex_165w	7	0.3	0.2	2.3
complex_191w	7	0.3	0.2	2.3
complex_161w	6	0.4	0.2	2.4
complex_175w	6	0.4	0.2	2.4
complex_183w	8	0.3	0.2	2.4
complex_180w	6	0.4	0.1	2.5
complex_174w	7	0.4	0.2	2.5
complex_172w	6	0.4	0.2	2.6
complex_179w	7	0.4	0.2	2.6
complex_160w	7	0.4	0.2	2.7
complex_158w	10	0.3	0.1	2.8
complex_176w	7	0.4	0.4	2.9
complex_159w	8	0.4	0.3	3.2
complex_169w	7	0.5	0.6	3.3
complex_166w	6	0.6	0.8	3.5
complex_154w	9	0.4	0.2	3.5
complex_171w	7	0.5	0.6	3.7
complex_170w	7	0.5	0.5	3.8
complex_95w	7	0.6	0.3	3.9
complex_163w	5	0.8	1.3	4.1
complex_148w	6	0.7	1.1	4.1
complex_49w	7	0.6	0.4	4.1
complex_177w	7	0.6	0.8	4.1

Violation Details for complex_167w (lowest sum total of violations)

Residue	Atom	Restraint Range (Å)	Model Distances (Å)	Violation Magnitude (Å)
V15A	H^N	3.8 - 6.8	9.0, 14.7, 7.2, 11.8	0.4
G67A	H^N	5.2 - 6.6	10.4, 15.4, 11.9, 6.9	0.3
S75A	H^N	5.0 - 10.6	11.0, 20.9, 17.2, 16.5	0.4
G9B	H^N	4.5 - 8.5	16.9, 8.7, 18.1, 16.1	0.2
T18B	H^N	7.0 - 8.0	16.6, 8.7, 10.9, 11.1	0.7
Y60B	H^N	4.1 - 7.2	7.5, 10.5, 15.4, 11.1	0.3

Violation Details for complex_163w (lowest number of violations)

Residue	Atom	Restraint Range (Å)	Model Distances (Å)	Violation Magnitude (Å)
V15A	H^N	3.8 – 6.8	11.7, 6.9, 15.6, 11.2	0.1
Y60A	H^N	3.6 - 6.8	6.9, 13.4, 13.1, 16.4	0.1
G67A	H^N	5.2 - 6.6	6.8, 11.9, 16.1, 12.6	0.2
S75A	H^N	5.0 - 10.6	16.3, 17.3, 22.5, 14.0	3.4
G9B	H^N	4.5 - 8.5	16.0, 18.3, 8.8, 14.8	0.3

Supplementary Table 4. Detailed H^N-F distance constraints between EmrE and F_4-TPP^+ used in the GROMACS refinement stage of structure calculation.

Constraint Atoms			Constr. Range		Constraint Atoms			Constr. Range	
Res.	Atom	Fluorine	r_0 (Å)	r_1 (Å)	Res.	Atom	Fluorine	r_0 (Å)	r_1 (Å)
G8A	HN	F3	7.7	40	Y6B	HN	F3	7.6	40
G8A	HN	F13	7.7	40	Y6B	HN	F13	7.6	40
G8A	HN	F18	7.7	40	Y6B	HN	F18	7.6	40
G8A	HN	F8	7.7	40	Y6B	HN	F8	7.6	40
G9A	HN	F3	8.2	40	G8B	HN	F3	7.8	40
G9A	HN	F13	8.2	40	G8B	HN	F13	7.8	40
G9A	HN	F18	8.2	40	G8B	HN	F18	7.8	40
G9A	HN	F8	8.2	40	G8B	HN	F8	7.8	40
A10A	HN	F3	8.1	40	G9B	HN	F13	4.5	8.5
A10A	HN	F13	8.1	40	A10B	HN	F3	7.2	40
A10A	HN	F18	8.1	40	A10B	HN	F13	7.2	40
A10A	HN	F8	8.1	40	A10B	HN	F18	7.2	40
I11A	HN	F3	5.3	40	A10B	HN	F8	7.2	40
I11A	HN	F13	5.3	40	I11B	HN	F3	5.3	40
I11A	HN	F18	5.3	40	I11B	HN	F13	5.3	40
I11A	HN	F8	5.3	40	I11B	HN	F18	5.3	40
A13A	HN	F3	7.4	40	I11B	HN	F8	5.3	40
A13A	HN	F13	7.4	40	G17B	HN	F13	4.8	9.4
A13A	HN	F18	7.4	40	T18B	HN	F13	7	8
A13A	HN	F8	7.4	40	T19B	HN	F3	8.1	40
E14A	HN	F8	5	9.6	T19B	HN	F13	8.1	40
V15A	HN	F8	3.8	6.8	T19B	HN	F18	8.1	40
I16A	HN	F3	7.7	40	T19B	HN	F8	8.1	40
I16A	HN	F13	7.7	40	K22B	HN	F3	8.2	40
I16A	HN	F18	7.7	40	K22B	HN	F13	8.2	40
I16A	HN	F8	7.7	40	K22B	HN	F18	8.2	40
G17A	HN	F8	4.5	8.3	K22B	HN	F8	8.2	40
T18A	HN	F8	6.9	9.9	E25B	HN	F3	8	40
T19A	HN	F3	6.4	40	E25B	HN	F13	8	40
T19A	HN	F13	6.4	40	E25B	HN	F18	8	40
T19A	HN	F18	6.4	40	E25B	HN	F8	8	40
T19A	HN	F8	6.4	40	G26B	HN	F3	7.9	40
L20A	HN	F3	6.6	40	G26B	HN	F13	7.9	40
L20A	HN	F13	6.6	40	G26B	HN	F18	7.9	40
L20A	HN	F18	6.6	40	G26B	HN	F8	7.9	40
L20A	HN	F8	6.6	40	R29B	HN	F3	7.8	40
M21A	HN	F3	6.7	40	R29B	HN	F13	7.8	40
M21A	HN	F13	6.7	40	R29B	HN	F18	7.8	40
M21A	HN	F18	6.7	40	R29B	HN	F8	7.8	40
M21A	HN	F8	6.7	40	L30B	HN	F3	8	40
K22A	HN	F3	8.2	40	L30B	HN	F13	8	40
K22A	HN	F13	8.2	40	L30B	HN	F18	8	40
K22A	HN	F18	8.2	40	L30B	HN	F8	8	40
K22A	HN	F8	8.2	40	W31B	HN	F3	7.7	40
F23A	HN	F3	8.2	40	W31B	HN	F13	7.7	40
F23A	HN	F13	8.2	40	W31B	HN	F18	7.7	40
F23A	HN	F18	8.2	40	W31B	HN	F8	7.7	40
F23A	HN	F8	8.2	40	S33B	HN	F3	7.8	40
S24A	HN	F3	6.9	40	S33B	HN	F13	7.8	40
S24A	HN	F13	6.9	40	S33B	HN	F18	7.8	40
S24A	HN	F18	6.9	40	S33B	HN	F8	7.8	40

S24A	HN	F8	6.9	40	V34B	HN	F3	8.1	40
E25A	HN	F3	8.2	40	V34B	HN	F13	8.1	40
E25A	HN	F13	8.2	40	V34B	HN	F18	8.1	40
E25A	HN	F18	8.2	40	V34B	HN	F8	8.1	40
E25A	HN	F8	8.2	40	G35B	HN	F3	8.2	40
G26A	HN	F3	8.2	40	G35B	HN	F13	8.2	40
G26A	HN	F13	8.2	40	G35B	HN	F18	8.2	40
G26A	HN	F18	8.2	40	G35B	HN	F8	8.2	40
G26A	HN	F8	8.2	40	S43B	HN	F13	4.2	7.7
F27A	HN	F3	8.2	40	L47B	HN	F3	5.2	40
F27A	HN	F13	8.2	40	L47B	HN	F13	5.2	40
F27A	HN	F18	8.2	40	L47B	HN	F18	5.2	40
F27A	HN	F8	8.2	40	L47B	HN	F8	5.2	40
R29A	HN	F3	8.1	40	Q49B	HN	F3	5.7	40
R29A	HN	F13	8.1	40	Q49B	HN	F13	5.7	40
R29A	HN	F18	8.1	40	Q49B	HN	F18	5.7	40
R29A	HN	F8	8.1	40	Q49B	HN	F8	5.7	40
L30A	HN	F3	8	40	L51B	HN	F3	7.5	40
L30A	HN	F13	8	40	L51B	HN	F13	7.5	40
L30A	HN	F18	8	40	L51B	HN	F18	7.5	40
L30A	HN	F8	8	40	L51B	HN	F8	7.5	40
W31A	HN	F3	8	40	A52B	HN	F3	8	40
W31A	HN	F13	8	40	A52B	HN	F13	8	40
W31A	HN	F18	8	40	A52B	HN	F18	8	40
W31A	HN	F8	8	40	A52B	HN	F8	8	40
S33A	HN	F3	7.8	40	Y53B	HN	F3	7.7	40
S33A	HN	F13	7.8	40	Y53B	HN	F13	7.7	40
S33A	HN	F18	7.8	40	Y53B	HN	F18	7.7	40
S33A	HN	F8	7.8	40	Y53B	HN	F8	7.7	40
V34A	HN	F3	8	40	G57B	HN	F3	8.1	40
V34A	HN	F13	8	40	G57B	HN	F13	8.1	40
V34A	HN	F18	8	40	G57B	HN	F18	8.1	40
V34A	HN	F8	8	40	G57B	HN	F8	8.1	40
G35A	HN	F3	7.1	40	I58B	HN	F3	8.1	40
G35A	HN	F13	7.1	40	I58B	HN	F13	8.1	40
G35A	HN	F18	7.1	40	I58B	HN	F18	8.1	40
G35A	HN	F8	7.1	40	I58B	HN	F8	8.1	40
C39A	HN	F3	5.5	40	A59B	HN	F3	7.6	40
C39A	HN	F13	5.5	40	A59B	HN	F13	7.6	40
C39A	HN	F18	5.5	40	A59B	HN	F18	7.6	40
C39A	HN	F8	5.5	40	A59B	HN	F8	7.6	40
Y40A	HN	F8	4.7	8.4	Y60B	HN	F18	4.1	7.2
C41A	HN	F3	6.1	40	A61B	HN	F3	6.8	40
C41A	HN	F13	6.1	40	A61B	HN	F13	6.8	40
C41A	HN	F18	6.1	40	A61B	HN	F18	6.8	40
C41A	HN	F8	6.1	40	A61B	HN	F8	6.8	40
S43A	HN	F8	2	5.6	I62B	HN	F3	5.7	40
F44A	HN	F8	3	5.6	I62B	HN	F13	5.7	40
Q49A	HN	F3	6.9	40	I62B	HN	F18	5.7	40
Q49A	HN	F13	6.9	40	I62B	HN	F8	5.7	40
Q49A	HN	F18	6.9	40	W63B	HN	F3	5.5	40
Q49A	HN	F8	6.9	40	W63B	HN	F13	5.5	40
G57A	HN	F3	5.1	40	W63B	HN	F18	5.5	40
G57A	HN	F13	5.1	40	W63B	HN	F8	5.5	40
G57A	HN	F18	5.1	40	V66B	HN	F3	6.3	40
G57A	HN	F8	5.1	40	V66B	HN	F13	6.3	40

I58A	HN	F3	8.1	40	V66B	HN	F18	6.3	40
I58A	HN	F13	8.1	40	V66B	HN	F8	6.3	40
I58A	HN	F18	8.1	40	G67B	HN	F3	7.5	40
I58A	HN	F8	8.1	40	G67B	HN	F13	7.5	40
A59A	HN	F3	5.7	40	G67B	HN	F18	7.5	40
A59A	HN	F13	5.7	40	G67B	HN	F8	7.5	40
A59A	HN	F18	5.7	40	I68B	HN	F3	6.5	40
A59A	HN	F8	5.7	40	I68B	HN	F13	6.5	40
Y60A	HN	F3	3.6	6.8	I68B	HN	F18	6.5	40
I62A	HN	F3	5.1	40	I68B	HN	F8	6.5	40
I62A	HN	F13	5.1	40	V69B	HN	F3	7.8	40
I62A	HN	F18	5.1	40	V69B	HN	F13	7.8	40
I62A	HN	F8	5.1	40	V69B	HN	F18	7.8	40
W63A	HN	F3	5.1	40	V69B	HN	F8	7.8	40
W63A	HN	F13	5.1	40	S75B	HN	F3	8.1	40
W63A	HN	F18	5.1	40	S75B	HN	F13	8.1	40
W63A	HN	F8	5.1	40	S75B	HN	F18	8.1	40
V64A	HN	F3	3.5	6.7	S75B	HN	F8	8.1	40
G65A	HN	F3	6.1	7.4	W76B	HN	F3	7.6	40
V66A	HN	F3	5.9	40	W76B	HN	F13	7.6	40
V66A	HN	F13	5.9	40	W76B	HN	F18	7.6	40
V66A	HN	F18	5.9	40	W76B	HN	F8	7.6	40
V66A	HN	F8	5.9	40	G77B	HN	F3	8.1	40
G67A	HN	F3	5.2	6.6	G77B	HN	F13	8.1	40
I68A	HN	F3	4.6	8.1	G77B	HN	F18	8.1	40
V69A	HN	F3	5.7	40	G77B	HN	F8	8.1	40
V69A	HN	F13	5.7	40	F78B	HN	F3	8.2	40
V69A	HN	F18	5.7	40	F78B	HN	F13	8.2	40
V69A	HN	F8	5.7	40	F78B	HN	F18	8.2	40
I71A	HN	F3	7.5	40	F78B	HN	F8	8.2	40
I71A	HN	F13	7.5	40	F79B	HN	F3	8.2	40
I71A	HN	F18	7.5	40	F79B	HN	F13	8.2	40
I71A	HN	F8	7.5	40	F79B	HN	F18	8.2	40
S72A	HN	F3	6.1	40	F79B	HN	F8	8.2	40
S72A	HN	F13	6.1	40	G80B	HN	F3	7.9	40
S72A	HN	F18	6.1	40	G80B	HN	F13	7.9	40
S72A	HN	F8	6.1	40	G80B	HN	F18	7.9	40
L74A	HN	F3	8	40	G80B	HN	F8	7.9	40
L74A	HN	F13	8	40	Q81B	HN	F3	7.8	40
L74A	HN	F18	8	40	Q81B	HN	F13	7.8	40
L74A	HN	F8	8	40	Q81B	HN	F18	7.8	40
S75A	HN	F18	5	10.6	Q81B	HN	F8	7.8	40
W76A	HN	F3	7.2	40	R82B	HN	F3	8.2	40
W76A	HN	F13	7.2	40	R82B	HN	F13	8.2	40
W76A	HN	F18	7.2	40	R82B	HN	F18	8.2	40
W76A	HN	F8	7.2	40	R82B	HN	F8	8.2	40
G77A	HN	F3	7.7	40	L83B	HN	F3	8	40
G77A	HN	F13	7.7	40	L83B	HN	F13	8	40
G77A	HN	F18	7.7	40	L83B	HN	F18	8	40
G77A	HN	F8	7.7	40	L83B	HN	F8	8	40
F79A	HN	F3	6.5	40	L85B	HN	F3	8.1	40
F79A	HN	F13	6.5	40	L85B	HN	F13	8.1	40
F79A	HN	F18	6.5	40	L85B	HN	F18	8.1	40
F79A	HN	F8	6.5	40	L85B	HN	F8	8.1	40
G80A	HN	F3	8	40	A87B	HN	F3	8.2	40
G80A	HN	F13	8	40	A87B	HN	F13	8.2	40

G80A	HN	F18	8	40
G80A	HN	F8	8	40
Q81A	HN	F3	8	40
Q81A	HN	F13	8	40
Q81A	HN	F18	8	40
Q81A	HN	F8	8	40
R82A	HN	F3	8.1	40
R82A	HN	F13	8.1	40
R82A	HN	F18	8.1	40
R82A	HN	F8	8.1	40
L83A	HN	F3	8	40
L83A	HN	F13	8	40
L83A	HN	F18	8	40
L83A	HN	F8	8	40
D84A	HN	F3	7.7	40
D84A	HN	F13	7.7	40
D84A	HN	F18	7.7	40
D84A	HN	F8	7.7	40
A87A	HN	F3	7.3	40
A87A	HN	F13	7.3	40
A87A	HN	F18	7.3	40
A87A	HN	F8	7.3	40
G90A	HN	F3	8	40
G90A	HN	F13	8	40
G90A	HN	F18	8	40
G90A	HN	F8	8	40
C95A	HN	F3	8	40
C95A	HN	F13	8	40
C95A	HN	F18	8	40
C95A	HN	F8	8	40
G97A	HN	F3	8.2	40
G97A	HN	F13	8.2	40
G97A	HN	F18	8.2	40
G97A	HN	F8	8.2	40
L103A	HN	F3	7.4	40
L103A	HN	F13	7.4	40
L103A	HN	F18	7.4	40
L103A	HN	F8	7.4	40
L104A	HN	F3	8.1	40
L104A	HN	F13	8.1	40
L104A	HN	F18	8.1	40
L104A	HN	F8	8.1	40
A87B	HN	F18	8.2	40
A87B	HN	F8	8.2	40
G90B	HN	F3	8.1	40
G90B	HN	F13	8.1	40
G90B	HN	F18	8.1	40
G90B	HN	F8	8.1	40
G97B	HN	F3	8.2	40
G97B	HN	F13	8.2	40
G97B	HN	F18	8.2	40
G97B	HN	F8	8.2	40
L103B	HN	F3	8.2	40
L103B	HN	F13	8.2	40
L103B	HN	F18	8.2	40
L103B	HN	F8	8.2	40
L104B	HN	F3	8.1	40
L104B	HN	F13	8.1	40
L104B	HN	F18	8.1	40
L104B	HN	F8	8.1	40
W63B	HE1	F13	0	5.8

Supplementary Table 5. Distance constraint violation statistics for the 10 lowest-violation structures from the GROMACS refinement stage of structure calculation and detailed violation analysis for two complexes selected from the two MD runs. Violation detail tables indicate distances from specified protein atoms to each of the four ligand fluorines, to score against the original four-fold ambiguous data set.

Ensemble Conformer #	MD Timepoint (ns)	MD Run #	Number of Violations	Average Violation (Å)	Std. Dev. Violation (Å)	Sum Total of Violations (Å)
1	280	2	4	0.2	0.1	0.8
2	200	2	5	0.3	0.2	1.5
3	210	2	5	0.3	0.3	1.7
4	335	2	4	0.4	0.2	1.7
5	330	1	4	0.4	0.2	1.8
6	335	1	4	0.5	0.3	1.8
7	355	1	5	0.4	0.1	1.8
8	260	2	3	0.6	0.2	1.9
9	385	1	4	0.5	0.2	1.9
10	315	1	5	0.4	0.2	1.9

Violation Details for Conformer 1 (Run 2, 280 ns timepoint)

Residue	Atom	Restraint Range (Å)	Model Distances (Å)	Violation Magnitude (Å)
V15A	H^N	3.8 - 6.8	10.9, 7.0, 15.0, 9.6	0.2
Y60A	H^N	3.6 – 6.8	6.9, 14.1, 13.9, 16.6	0.1
G9B	H^N	4.5 – 8.5	16.8, 18.4, 8.9, 14.8	0.4
T18B	H^N	7.0 – 8.0	10.8, 13.7, 8.1, 16.6	0.1

Violation Details for Conformer 5 (Run 1, 330 ns timepoint)

Residue	Atom	Restraint Range (Å)	Model Distances (Å)	Violation Magnitude (Å)
V15A	H^N	3.8 - 6.8	12.6, 16.8, 7.3, 13.5	0.5
Y60A	H^N	3.6 – 6.8	17.0, 14.4, 13.4, 7.0	0.2
G9B	H^N	4.5 – 8.5	15.1, 9.2, 18.3, 17.7	0.7
T18B	H^N	7.0 – 8.0	17.8, 8.4, 13.7, 13.6	0.4

Supplementary Table 6. Detailed parameters for the solid-state NMR experiments for resonance assignment and distance measurements of F_4-TPP^+ bound CDN-labeled EmrE in DMPC bilayers at pH 8.0. The protein : lipid molar ratio is 1 : 25.

Experiment	NMR Parameters	Expt. Time
$^1H^{15}N$ solution TROSY-HSQC	B_0 = 17.6 T; T_{eff} = 318 K; ns = 16 (32 for pH 8.4); d1 = 2 s	2 hr * 3+ 4 hr = 10 hr
2D hNH	B_0 = 14.1 T; 1.3 mm HCN; T_{eff} = 280 K (T_{set} = 248 K); ν_{MAS} = 55 kHz; ns = 48; τ_{rd} = 1.2 s; $\tau_{CP\text{-}HN}$ = 1.2 ms; $\nu_{1H\text{-}CP,HN}$ = 93.5 kHz; $\nu_{15N\text{-}CP,HN}$ = 38.5 kHz; $t_{1,max}$ = 45 ms; $t_{1,inc}$ = 300.0 μs; $\nu_{1H\text{-}DD,t1evol}$ = 10 kHz; τ_{solsup} = 200 ms; $\nu_{1H\text{-}solsup}$ = 15 kHz; $\tau_{CP\text{-}NH}$ = 1.0 ms; $\nu_{1H\text{-}CP,NH}$ = 93.5 kHz; $\nu_{15N\text{-}CP,NH}$ = 38.5 kHz; τ_{dwell} = 10.0 μs; τ_{acq} = 20.48; $\nu_{15N\text{-}DD,acq}$ = 10 kHz; $\nu_{13C\text{-}DD,acq}$ = 10 kHz	6 hr
3D hCANH	B_0 = 14.1 T; 1.3 mm HCN; T_{eff} = 280 K (T_{set} = 248 K); ν_{MAS} = 55 kHz; ns = 24 (8 * 3 blocks); τ_{rd} = 1.8 s; $\tau_{CP\text{-}HC}$ = 1.1 ms; $\nu_{1H\text{-}CP,HC}$ = 90.0 kHz; $\nu_{13C\text{-}CP,HC}$ = 35 kHz; $t_{1,max}$ = 4.32 ms; $t_{1,inc}$ = 160.0 μs; $\nu_{1H\text{-}DD,\ t1evol}$ = 10 kHz; $\tau_{DCP\text{-}CN}$ = 7.4 ms; $\nu_{13C\text{-}DCP}$ = 35.0 kHz; $\nu_{15N\text{-}DCP}$ = 20.0 kHz; $\nu_{1H\text{-}DD,DCP}$ = 10 kHz; $t_{2,max}$ = 10.5 ms; $t_{2,inc}$ = 300 μs; $\nu_{1H\text{-}DD,\ t2evol}$ = 10 kHz; τ_{solsup} = 200 ms; $\nu_{1H\text{-}solsup}$ = 15 kHz; $\tau_{CP\text{-}NH}$ = 1.0 ms; $\nu_{1H\text{-}CP,NH}$ = 93.5 kHz; $\nu_{15N\text{-}CP,NH}$ = 38.5 kHz; τ_{dwell} = 10.0 μs; τ_{acq} = 20.48 ms; $\nu_{15N\text{-}DD,acq}$ = 10 kHz; $\nu_{13C\text{-}DD,acq}$ = 10 kHz	17 hr* 3 = 51 hr
3D hCOcaNH	B_0 = 14.1 T; 1.3 mm HCN; T_{eff} = 280 K (T_{set} = 248 K); ν_{MAS} = 55 kHz; ns = 40 (16 + 24); τ_{rd} = 1.4 s; $\tau_{CP\text{-}HC}$ = 1.4 ms; $\nu_{1H\text{-}CP,HC}$ = 93.5 kHz; $\nu_{13C\text{-}CP,HC}$ = 38.5 kHz; $t_{1,max}$ = 5.0 ms; $t_{1,inc}$ = 250.0 μs; $\nu_{1H\text{-}DD,\ t1evol}$ = 10 kHz; τ_{DREAM} = 6.5 ms; $\tau_{DCP\text{-}CN}$ = 5.0 ms; $\nu_{13C\text{-}DCP}$ = 20.0 kHz; $\nu_{15N\text{-}DCP}$ = 35.0 kHz; $\nu_{1H\text{-}DD,DCP}$ = 10 kHz; $t_{2,max}$ = 10.5 ms; $t_{2,inc}$ = 300.0 μs; $\nu_{1H\text{-}DD,\ t2evol}$ = 10 kHz; τ_{solsup} = 200 ms; $\nu_{1H\text{-}solsup}$ = 15 kHz; $\tau_{CP\text{-}NH}$ = 0.70 ms; $\nu_{1H\text{-}CP,NH}$ = 93.5 kHz; $\nu_{15N\text{-}CP,NH}$ = 38.5 kHz; τ_{dwell} = 10.0 μs; τ_{acq} = 20.48 ms; $\nu_{15N\text{-}DD,acq}$ = 10 kHz; $\nu_{13C\text{-}DD,acq}$ = 10 kHz	20 + 30 = 50 hr
3D hCONH	B_0 = 14.1 T; 1.3 mm HCN; T_{eff} = 280 K (T_{set} = 248 K); ν_{MAS} = 55 kHz; ns = 16; τ_{rd} = 1.2 s; $\tau_{CP\text{-}HC}$ = 1.5 ms; $\nu_{1H\text{-}CP,HC}$ = 93.5 kHz; $\nu_{13C\text{-}CP,HC}$ = 38.5 kHz; $t_{1,max}$ = 5.0 ms; $t_{1,inc}$ = 250.0 μs; $\nu_{1H\text{-}DD,\ t1evol}$ = 10 kHz; $\tau_{DCP\text{-}CN}$ = 7.1 ms; $\nu_{13C\text{-}DCP}$ = 35.0 kHz; $\nu_{15N\text{-}DCP}$ = 20.0 kHz; $\nu_{1H\text{-}DD,DCP}$ = 10 kHz; $t_{2,max}$ = 10.5 ms; $t_{2,inc}$ = 300.0 μs; $\nu_{1H\text{-}DD,\ t2evol}$ = 10 kHz; τ_{solsup} = 200 ms; $\nu_{1H\text{-}solsup}$ = 15 kHz; $\tau_{CP\text{-}NH}$ = 0.8 ms; $\nu_{1H\text{-}CP,NH}$ = 93.5 kHz; $\nu_{15N\text{-}CP,NH}$ = 38.5 kHz; τ_{dwell} = 10.0 μs; τ_{acq} = 20.48 ms; $\nu_{15N\text{-}DD,acq}$ = 10 kHz; $\nu_{13C\text{-}DD,acq}$ = 10 kHz	17.4 hr
3D hCAcoNH	B_0 = 14.1 T; 1.3 mm HCN; T_{eff} = 280 K (T_{set} = 245 K); ν_{MAS} = 55 kHz; ns = 32 (16 * 2 blocks); τ_{rd} = 1.4 s; $\tau_{CP\text{-}HC}$ = 1.0 ms; $\nu_{1H\text{-}CP,HC}$ = 93.5 kHz; $\nu_{13C\text{-}CP,HC}$ = 38.5 kHz; $t_{1,max}$ = 4.32 ms; $t_{1,inc}$ = 160.0 μs; $\nu_{1H\text{-}DD,\ t1evol}$ = 10 kHz; τ_{DREAM} = 5.0 ms; $\tau_{DCP\text{-}CN}$ = 6.0 ms; $\nu_{13C\text{-}DCP}$ = 35.0 kHz; $\nu_{15N\text{-}DCP}$ = 20.0 kHz; $\nu_{1H\text{-}DD,DCP}$ = 10 kHz; $t_{2,max}$ = 10.5 ms; $t_{2,inc}$ = 300.0 μs; $\nu_{1H\text{-}DD,\ t2evol}$ = 10 kHz; τ_{solsup} = 200 ms; $\nu_{1H\text{-}solsup}$ = 15 kHz; $\tau_{CP\text{-}NH}$ = 1.1 ms; $\nu_{1H\text{-}CP,NH}$ = 93.5 kHz; $\nu_{15N\text{-}CP,NH}$ = 38.5 kHz; τ_{dwell} = 10.0 μs; τ_{acq} = 20.48 ms; $\nu_{15N\text{-}DD,acq}$ = 10 kHz; $\nu_{13C\text{-}DD,acq}$ = 10 kHz	2 * 27 hr = 54 hr
3D hcaCBcaNH	B_0 = 14.1 T; 1.3 mm HCN; T_{eff} = 280 K (T_{set} = 245 K); ν_{MAS} = 55 kHz; ns = 24 (8 * 3 blocks); τ_{rd} = 1.2 s; $\tau_{CP\text{-}HC}$ = 1.1 ms; $\nu_{1H\text{-}CP,HC}$ = 93.5 kHz; $\nu_{13C\text{-}CP,HC}$ = 38.5 kHz; $t_{1,max}$ = 4.48 ms; $t_{1,inc}$ = 102.0 μs; $\nu_{1H\text{-}DD,\ t1evol}$ = 10 kHz; τ_{INEPT} = 6.5 ms * 2; τ_{ZF} = 3.0 ms; $\tau_{DCP\text{-}CN}$ = 7.8 ms; $\nu_{13C\text{-}DCP}$ = 30.0 kHz; $\nu_{15N\text{-}DCP}$ = 25.0 kHz; $t_{2,max}$ = 10.5 ms; $t_{2,inc}$ = 300.0 μs; $\nu_{1H\text{-}DD,\ t2evol}$ = 10 kHz; τ_{solsup} = 200 ms; $\nu_{1H\text{-}solsup}$ = 15 kHz; $\tau_{CP\text{-}NH}$ = 1.2 ms; $\nu_{1H\text{-}CP,NH}$ = 93.5 kHz; $\nu_{15N\text{-}CP,NH}$ = 38.5 kHz; τ_{dwell} = 10.0 μs; τ_{acq} = 20.48 ms; $\nu_{15N\text{-}DD,acq}$ = 10 kHz; $\nu_{13C\text{-}DD,acq}$ = 10 kHz	3 * 19 hr = 57 hr
3D hcaCBcacoNH	B_0 = 14.1 T; 1.3 mm HCN; T_{eff} = 280 K (T_{set} = 245 K); ν_{MAS} = 55 kHz; ns = 32 (16 * 2 blocks); τ_{rd} = 1.5 s; $\tau_{CP\text{-}HC}$ = 1.1 ms; $\nu_{1H\text{-}CP,HC}$ = 93.5 kHz; $\nu_{13C\text{-}CP,HC}$ = 38.5 kHz; $t_{1,max}$ = 4.48 ms; $t_{1,inc}$ = 102.0 μs; $\nu_{1H\text{-}DD,\ t1evol}$ = 10 kHz; τ_{INEPT} = 6.5 ms * 2; τ_{ZF} = 3.0 ms; τ_{DREAM} = 3.8 ms; $\tau_{DCP\text{-}CN}$ = 6.0 ms; $\nu_{13C\text{-}DCP}$ = 30.0 kHz; $\nu_{15N\text{-}DCP}$ = 25.0 kHz; $t_{2,max}$ = 10.5 ms; $t_{2,inc}$ = 300.0 μs; $\nu_{1H\text{-}DD,\ t2evol}$ = 10 kHz; τ_{solsup} = 200 ms; $\nu_{1H\text{-}solsup}$ = 15 kHz; $\tau_{CP\text{-}NH}$ = 1.2 ms; $\nu_{1H\text{-}CP,NH}$ = 93.5 kHz; $\nu_{15N\text{-}CP,NH}$ = 38.5 kHz; τ_{dwell} = 10.0 μs; τ_{acq} = 20.48 ms; $\nu_{15N\text{-}DD,acq}$ = 10 kHz; $\nu_{13C\text{-}DD,acq}$ = 10 kHz	2 * 46.5 hr = 93 hr
2D ^{19}F-^{19}F exchange, (Intermediate T)	B_0 = 14.1 T; 1.9 mm HFX; T_{eff} = 285 K (T_{set} = 250 K); ν_{MAS} = 38 kHz; τ_{rd} = 2.0 s; $t_{1,max}$ = 1.579 ms; $t_{1,inc}$ = 78.95 μs; $\nu_{1H\text{-}DD,\ t1evol}$ = 10 kHz; τ_{dwell} = 2.5 μs; τ_{acq} = 7.68 ms; $\nu_{1H\text{-}DD,acq}$ = 10 kHz; ns = [256, 256, 256, 256, 384, 512, 256], τ_{mix} = [0.1, 1.0, 5.0, 10.0, 20.0, 40.0, 75.0] ms	5.7 hr per ns = 256 block, total 48.5 hr
2D H-N H^N-F REDOR, S_0 and S pair	B_0 = 14.1 T; 1.9 mm HFX; T_{eff} = 282 K (T_{set} = 243 K); ν_{MAS} = 38 kHz; τ_{rd} = 2.0 s; $\tau_{CP\text{-}HN}$ = 0.7 ms; $\nu_{1H\text{-}CP,HN}$ = 88.0 kHz; $\nu_{15N\text{-}CP,HN}$ = 50.0 kHz; $t_{1,max}$ = 28 ms; $t_{1,inc}$ = 200.0 μs; $\nu_{1H\text{-}DD,\ t1evol}$ = 10 kHz; τ_{solsup} = 200 ms; $\nu_{1H\text{-}solsup}$ = 15 kHz; $\tau_{CP\text{-}}$	31.5 * 2 = 63 hr, 28.7 * 2 = 57.5 hr, 32.9 * 2 = 65.7 hr

	$_{HN}$ = 0.7 ms; $\nu_{1H\text{-}CP,NH}$ = 88.0 kHz; $\nu_{15N\text{-}CP,HN}$ = 50.0 kHz; $\nu_{19F,pulse}$ = 83.3 kHz; $\nu_{15N\text{-}DD, REDOR}$ = 10 kHz; τ_{dwell} = 15.0 μs; τ_{acq} = 30.7 ms; $\nu_{15N\text{-}DD,acq}$ = 10 kHz, ns (S and S_0 each) = [56+64+64 = 184, 40+64+64 = 168, 128+64 = 192], $N\tau_{r,REDOR}$ = [64, 96, 144], $\tau_{mix, REDOR}$ = [1.68, 2.52, 3.78] ms	Total = 186 hr

Symbols: B_0 = magnetic field; NMR probe (rotor diameter, channels); T_{eff} = effective sample temperature; T_{set} = thermocouple-reported bearing gas temperature; ν_{MAS} = MAS frequency; ns = number of scans (transients) per free induction decay (FID); τ_{rd} = recycle delay between scans; $t_{1,max}$ = maximum t_1 (indirect dimension 1) evolution time; $t_{1,inc}$ = increment for t_1 (indirect dimension 1) evolution time; $t_{2,max}$ = maximum t_2 (indirect dimension 2) evolution time; $t_{2,inc}$ = increment for t_2 (indirect dimension 2) evolution time; τ_{dwell} = dwell time during direct FID acquisition; τ_{acq} = maximum acquisition time during direct FID detection; $\tau_{CP\text{-}XY}$ = cross polarization (CP) contact time during CP *from* channel X *to* channel Y; $\nu_{nuc\text{-}CP,XY}$ = radiofrequency field strength for CP spin lock on *nuc* (*nuc* = ^{1}H, ^{13}C, ^{15}N, or ^{19}F) during CP from X to Y; $\nu_{1H\text{-}DD, t1evol}$ = ^{1}H dipolar decoupling field strength during t_1 evolution; $\nu_{1H\text{-}DD, t2evol}$ = ^{1}H dipolar decoupling field strength during t_2 evolution; $\nu_{nuc\text{-}DD, acq}$ = dipolar decoupling field strength during FID acquisition on channel *nuc*; $\tau_{DCP\text{-}XY}$ = CP contact time during heteronuclear (specific / double) CP from channel X to channel Y; $\nu_{nuc\text{-}DCP}$ = radiofrequency spin lock field strength on *nuc* during heteronuclear CP; $\nu_{1H\text{-}DD,DCP}$ = ^{1}H dipolar decoupling field strength during heteronuclear CP; τ_{DREAM} = spin lock contact time during homonuclear coherence transfer with the DREAM condition; τ_{INEPT} = delay for antiphase coherence evolution/reconversion for Ca-Cb INEPT; τ_{ZF} = post-INEPT Z-filter time for removal of unwanted coherences; τ_{solsup} = solvent suppression mixing time during MISSISIPPI period; $\nu_{1H\text{-}solsup}$ = radiofrequency field strength during solvent suppression; $N_{N\tau r,REDOR}$ = number of rotor periods of REDOR recoupling; $\nu_{19F,pulse}$ = ^{19}F pulse field strength during REDOR recoupling; $\nu_{15N\text{-}DD, REDOR}$ = ^{15}N dipolar decoupling field strength during REDOR recoupling.

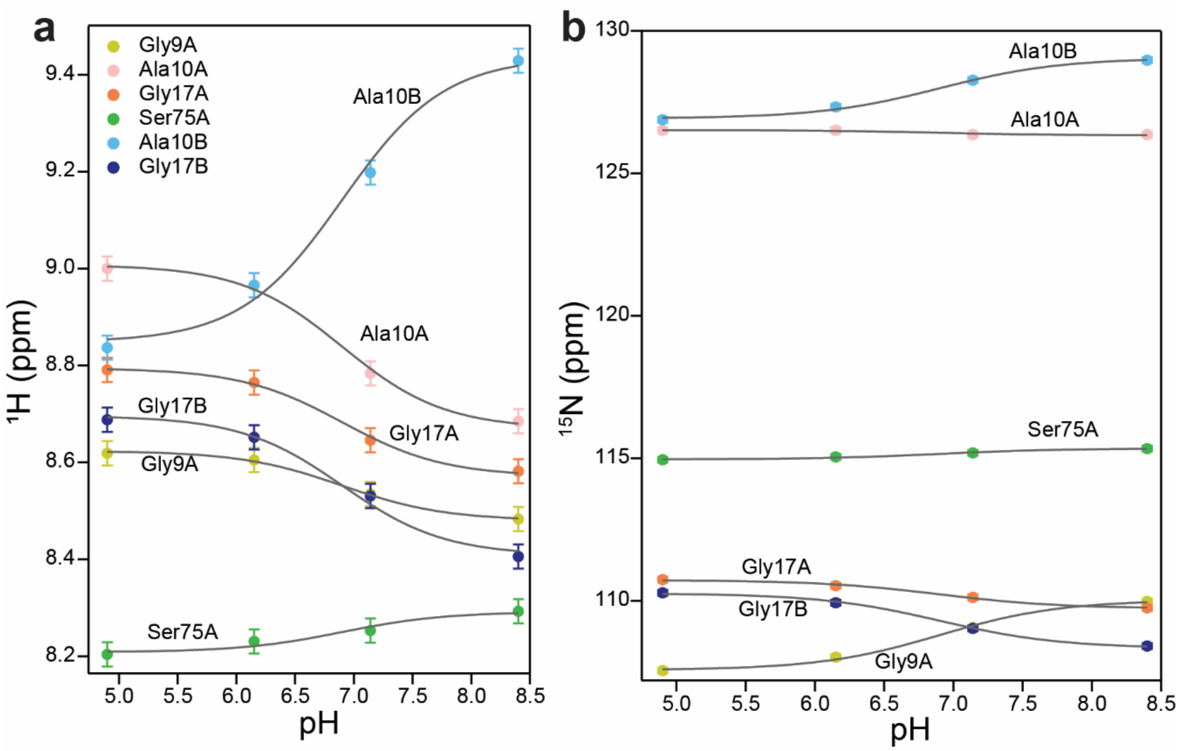

Supplementary Figure 1. pK_a determination of F_4-TPP^+-bound S64V-EmrE by solution NMR in DMPC/DHPC bicelles. **(a)** ^1H chemical shifts for six well resolved residues as a function of pH. **(b)** ^{15}N chemical shifts for the same residues as a function of pH. Global fits (grey lines) were calculated, yielding a pK_a value of 6.9 ± 0.1. The pH-dependent ^1H and ^{15}N chemical shifts are provided as a Source Data file.

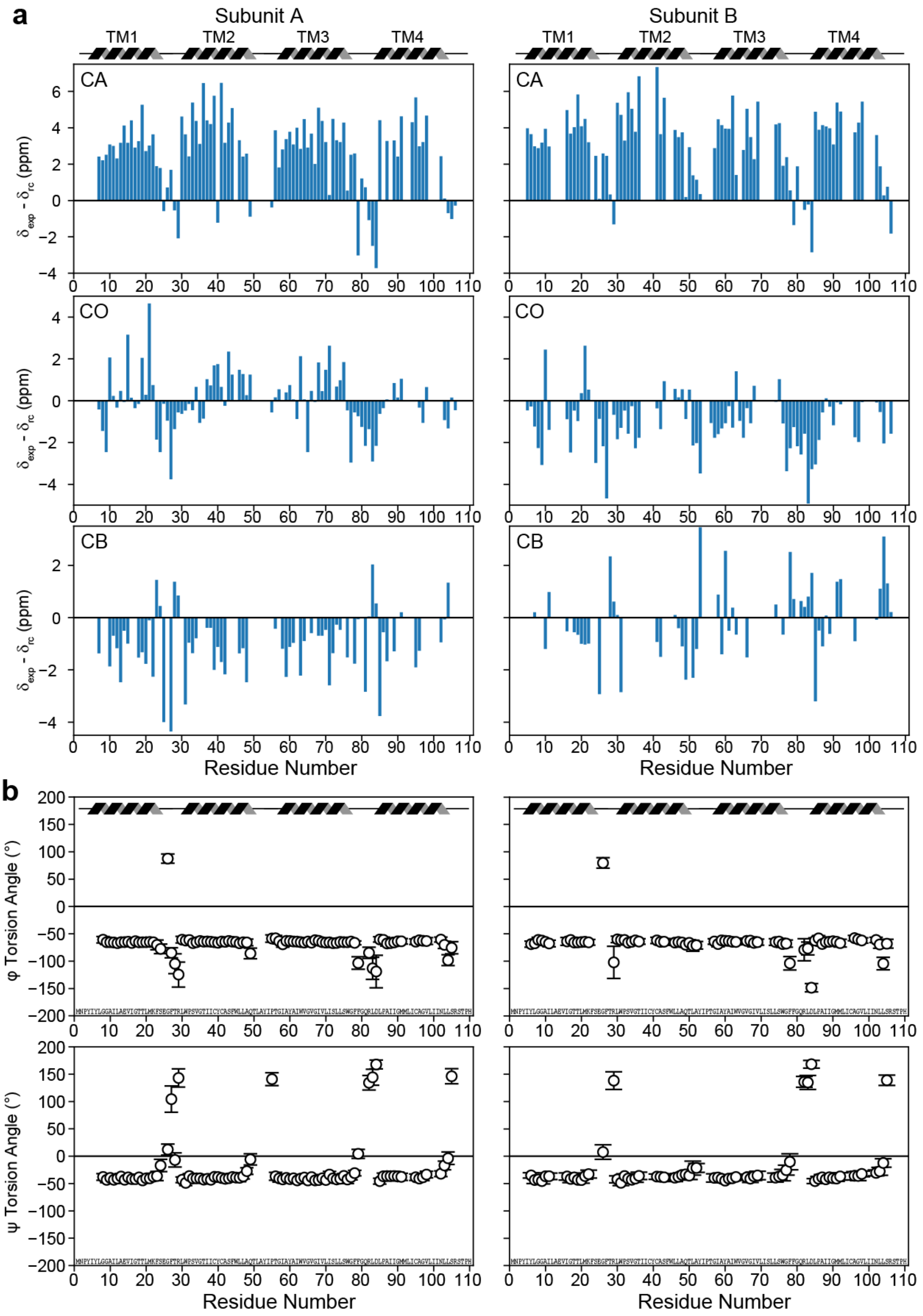

Supplementary Figure 2. Secondary structure of the pH 8.0 EmrE-TPP complex in DMPC bilayers derived from chemical shifts. (**a**) Secondary chemical shifts of Cα, Cβ, CO of EmrE at pH 8.0. (**b**) TALOS

predictions of Phi/Psi torsion angles. Errors for torsion angles are directly from TALOS output and represent 1σ error.

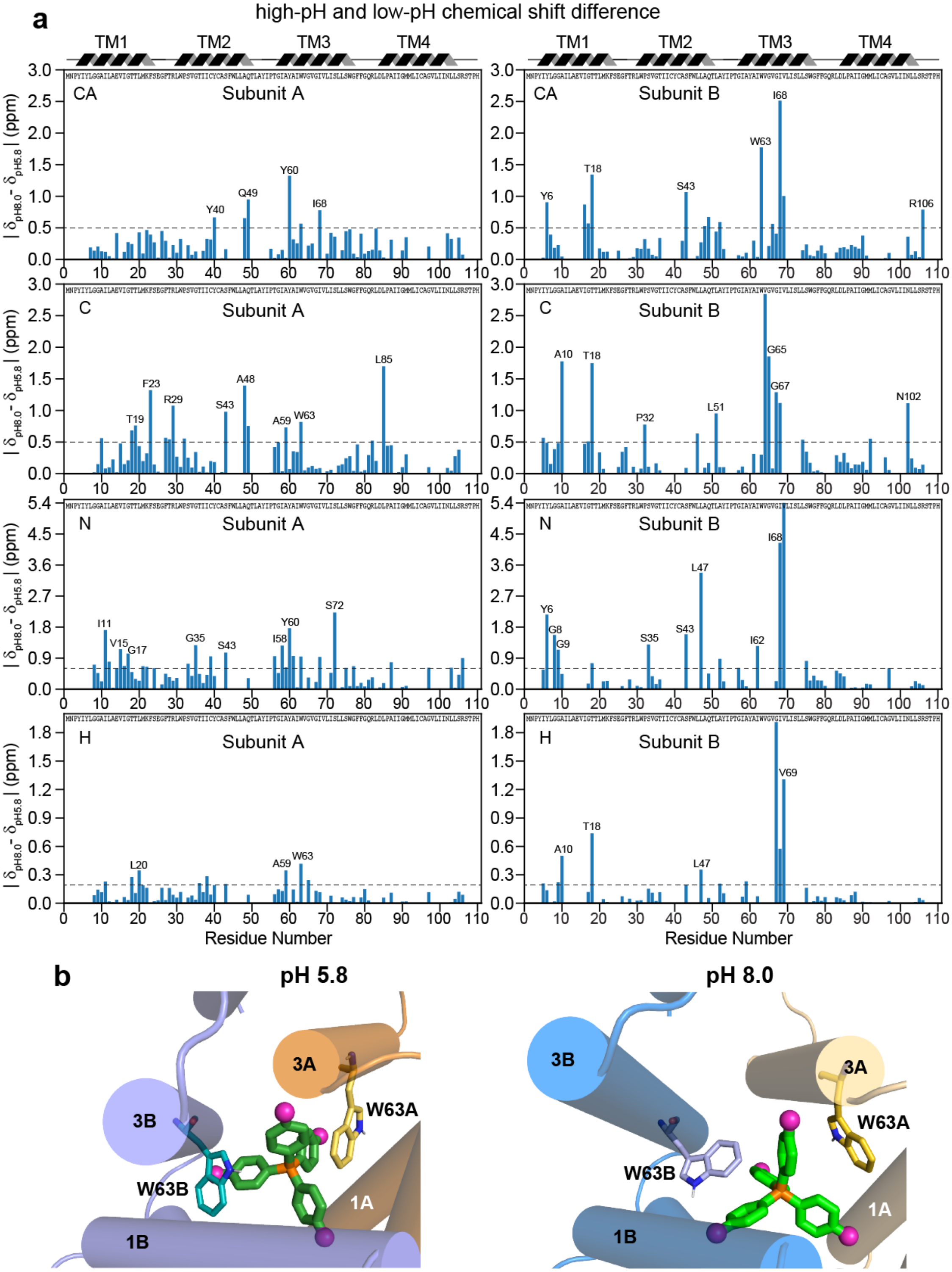

Supplementary Figure 3. Chemical shift differences of EmrE backbone atoms between pH 8.0 and pH 5.8. (**a**) CA, CO, N, and H chemical shift differences between pH 8.0 and pH 5.8. (**b**) The F_4-TPP^+ binding pocket in the low-pH and high-pH complex. The W63B indole ring is oriented differently with respect to the TM3B helix between the two structures, while the W63A indole has similar orientation relative to TM3A at both pH.

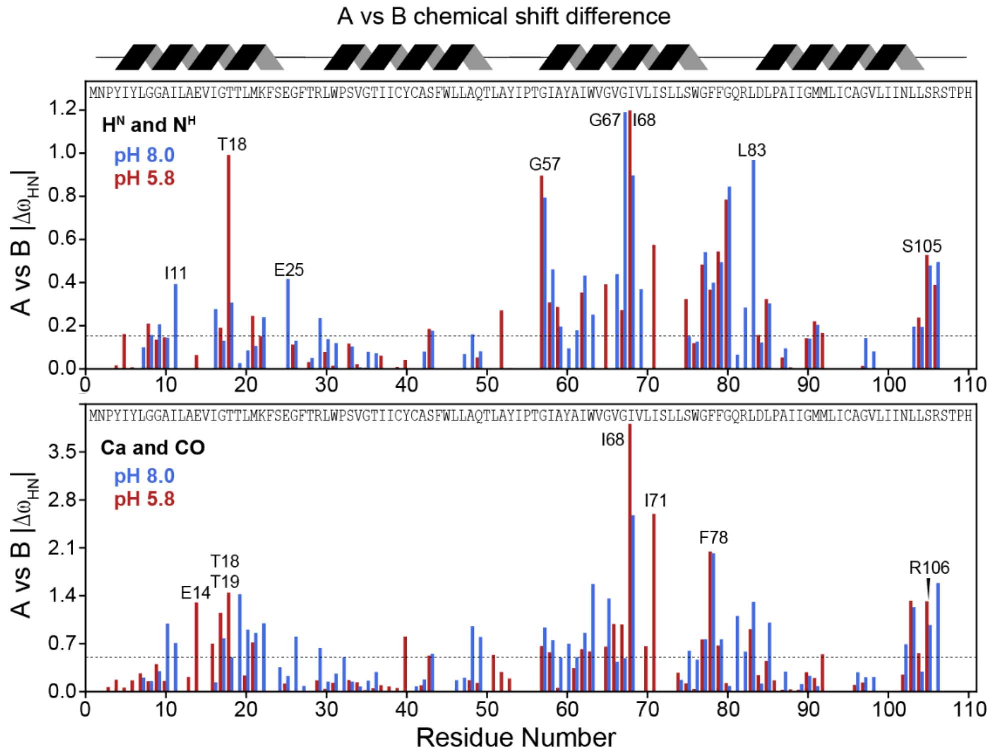

Supplementary Figure 4. Conformational asymmetry between subunits A and B of EmrE in DMPC bilayers. (**a**) Composite H^N and ^{15}N chemical shift differences between the two subunits. (**b**) Composite $C\alpha$ and CO chemical shift differences between the two subunits. Dashed lines indicate the linewidth-based estimate of the significance levels for chemical shift changes, which are 0.15 ppm for the composite amide and 0.5 ppm for ^{13}C.

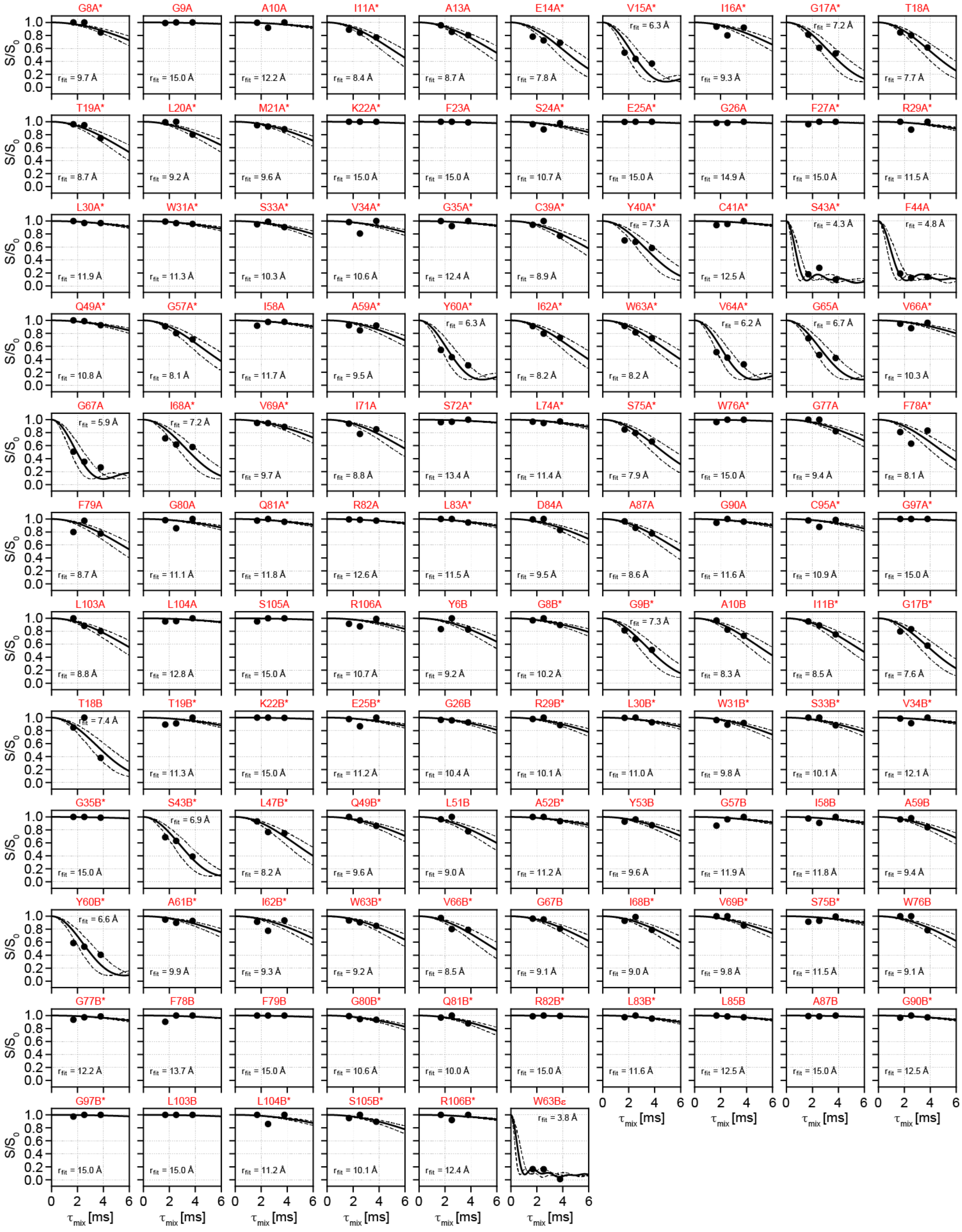
S/S$_0$
1.0
0.8
0.6
0.4
0.2
0.0
0
2
4
6
τ_{mix} [ms]
G8A* r_{fit} = 9.7 Å
G9A r_{fit} = 15.0 Å
A10A r_{fit} = 12.2 Å
I11A* r_{fit} = 8.4 Å
A13A r_{fit} = 8.7 Å
E14A* r_{fit} = 7.8 Å
V15A* r_{fit} = 6.3 Å
I16A* r_{fit} = 9.3 Å
G17A* r_{fit} = 7.2 Å
T18A r_{fit} = 7.7 Å
T19A* r_{fit} = 8.7 Å
L20A* r_{fit} = 9.2 Å
M21A* r_{fit} = 9.6 Å
K22A* r_{fit} = 15.0 Å
F23A r_{fit} = 15.0 Å
S24A* r_{fit} = 10.7 Å
E25A* r_{fit} = 15.0 Å
G26A r_{fit} = 14.9 Å
F27A* r_{fit} = 15.0 Å
R29A* r_{fit} = 11.5 Å
L30A* r_{fit} = 11.9 Å
W31A* r_{fit} = 11.3 Å
S33A* r_{fit} = 10.3 Å
V34A* r_{fit} = 10.6 Å
G35A* r_{fit} = 12.4 Å
C39A* r_{fit} = 8.9 Å
Y40A* r_{fit} = 7.3 Å
C41A* r_{fit} = 12.5 Å
S43A* r_{fit} = 4.3 Å
F44A r_{fit} = 4.8 Å
Q49A* r_{fit} = 10.8 Å
G57A* r_{fit} = 8.1 Å
I58A r_{fit} = 11.7 Å
A59A* r_{fit} = 9.5 Å
Y60A* r_{fit} = 6.3 Å
I62A* r_{fit} = 8.2 Å
W63A* r_{fit} = 8.2 Å
V64A* r_{fit} = 6.2 Å
G65A r_{fit} = 6.7 Å
V66A* r_{fit} = 10.3 Å
G67A r_{fit} = 5.9 Å
I68A* r_{fit} = 7.2 Å
V69A* r_{fit} = 9.7 Å
I71A r_{fit} = 8.8 Å
S72A* r_{fit} = 13.4 Å
L74A* r_{fit} = 11.4 Å
S75A* r_{fit} = 7.9 Å
W76A* r_{fit} = 15.0 Å
G77A r_{fit} = 9.4 Å
F78A* r_{fit} = 8.1 Å
F79A r_{fit} = 8.7 Å
G80A r_{fit} = 11.1 Å
Q81A* r_{fit} = 11.8 Å
R82A r_{fit} = 12.6 Å
L83A* r_{fit} = 11.5 Å
D84A r_{fit} = 9.5 Å
A87A r_{fit} = 8.6 Å
G90A r_{fit} = 11.6 Å
C95A* r_{fit} = 10.9 Å
G97A* r_{fit} = 15.0 Å
L103A r_{fit} = 8.8 Å
L104A r_{fit} = 12.8 Å
S105A r_{fit} = 15.0 Å
R106A r_{fit} = 10.7 Å
Y6B r_{fit} = 9.2 Å
G8B* r_{fit} = 10.2 Å
G9B* r_{fit} = 7.3 Å
A10B r_{fit} = 8.3 Å
I11B* r_{fit} = 8.5 Å
G17B* r_{fit} = 7.6 Å
T18B r_{fit} = 7.4 Å
T19B* r_{fit} = 11.3 Å
K22B* r_{fit} = 15.0 Å
E25B* r_{fit} = 11.2 Å
G26B r_{fit} = 10.4 Å
R29B* r_{fit} = 10.1 Å
L30B* r_{fit} = 11.0 Å
W31B* r_{fit} = 9.8 Å
S33B* r_{fit} = 10.1 Å
V34B* r_{fit} = 12.1 Å
G35B* r_{fit} = 15.0 Å
S43B* r_{fit} = 6.9 Å
L47B* r_{fit} = 8.2 Å
Q49B* r_{fit} = 9.6 Å
L51B r_{fit} = 9.0 Å
A52B* r_{fit} = 11.2 Å
Y53B r_{fit} = 9.6 Å
G57B r_{fit} = 11.9 Å
I58B r_{fit} = 11.8 Å
A59B r_{fit} = 9.4 Å
Y60B* r_{fit} = 6.6 Å
A61B* r_{fit} = 9.9 Å
I62B* r_{fit} = 9.3 Å
W63B* r_{fit} = 9.2 Å
V66B* r_{fit} = 8.5 Å
G67B r_{fit} = 9.1 Å
I68B* r_{fit} = 9.0 Å
V69B* r_{fit} = 9.8 Å
S75B* r_{fit} = 11.5 Å
W76B r_{fit} = 9.1 Å
G77B* r_{fit} = 12.2 Å
F78B r_{fit} = 13.7 Å
F79B r_{fit} = 15.0 Å
G80B* r_{fit} = 10.6 Å
Q81B* r_{fit} = 10.0 Å
R82B* r_{fit} = 15.0 Å
L83B* r_{fit} = 11.6 Å
L85B r_{fit} = 12.5 Å
A87B r_{fit} = 15.0 Å
G90B* r_{fit} = 12.5 Å
G97B* r_{fit} = 15.0 Å
L103B r_{fit} = 15.0 Å
L104B* r_{fit} = 11.2 Å
S105B* r_{fit} = 10.1 Å
R106B* r_{fit} = 12.4 Å
W63Bε r_{fit} = 3.8 Å

Supplementary Figure 5. All resolved ^1H-^{19}F REDOR dephasing curves for the EmrE-TPP complex at pH 8.0 in DMPC bilayers. For each resolved H^N peak, the simulated REDOR curve for the best-fit distance is overlaid with the experimental data. Asterisks indicate residues whose signals are partially overlapped in the 2D spectra.

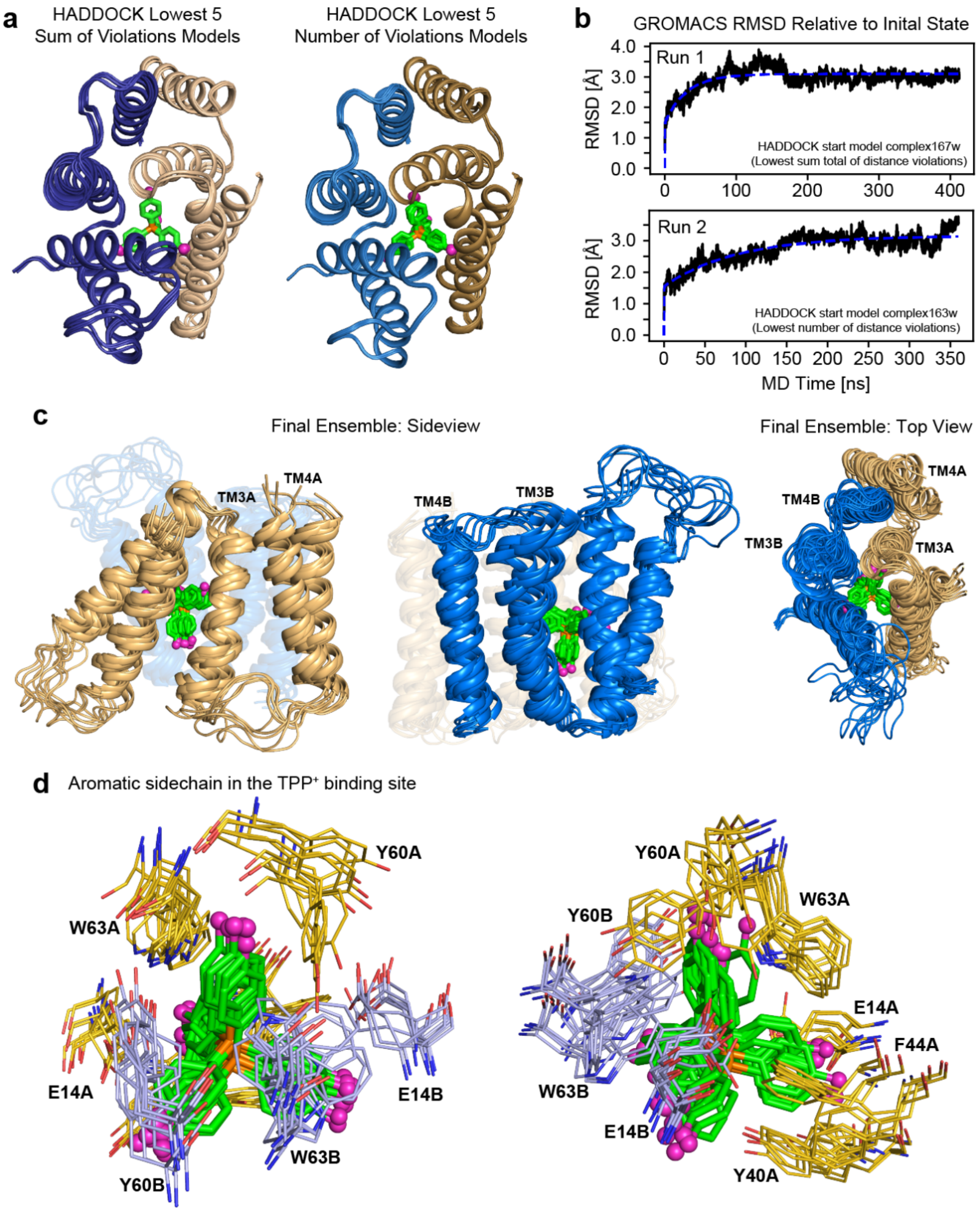

Supplementary Figure 6. Structure calculation of the pH 8.0 EmrE-TPP complex. (**a**) HADDOCK result of docking F_4-TPP^+ into the pH 5.8 structure with the distance constraints obtained at pH 8.0. The left view shows the 5 best models scored by the lowest sum of distance restraint violations (mean violation magnitude 2.2 ± 0.2 Å, mean number of violations = 8.4 ± 0.5). The right view shows the 5 best models scored by the lowest number of violations (mean violation magnitude 2.4 ± 0.3 Å, mean number of violations = 7.8 ± 0.4). The ligand center P atom has an RMSD of 0.2 ± 0.1 Å for the left ensemble and 0.5 ± 0.3 Å for the right ensemble. (**b**) Gromacs MD trajectory RMSD over 350-400 ns compared to the initial state. MD is equilibrated by 200 ns for both runs. (**c**) Side views and top view of the pH 8.0 EmrE-TPP ensemble. (**d**) Important binding-site sidechains (E14, Y40, F44, Y60 and W63) in the ensemble of 10 structures. Left view and right view differ by a ~180˚ rotation about the membrane normal.

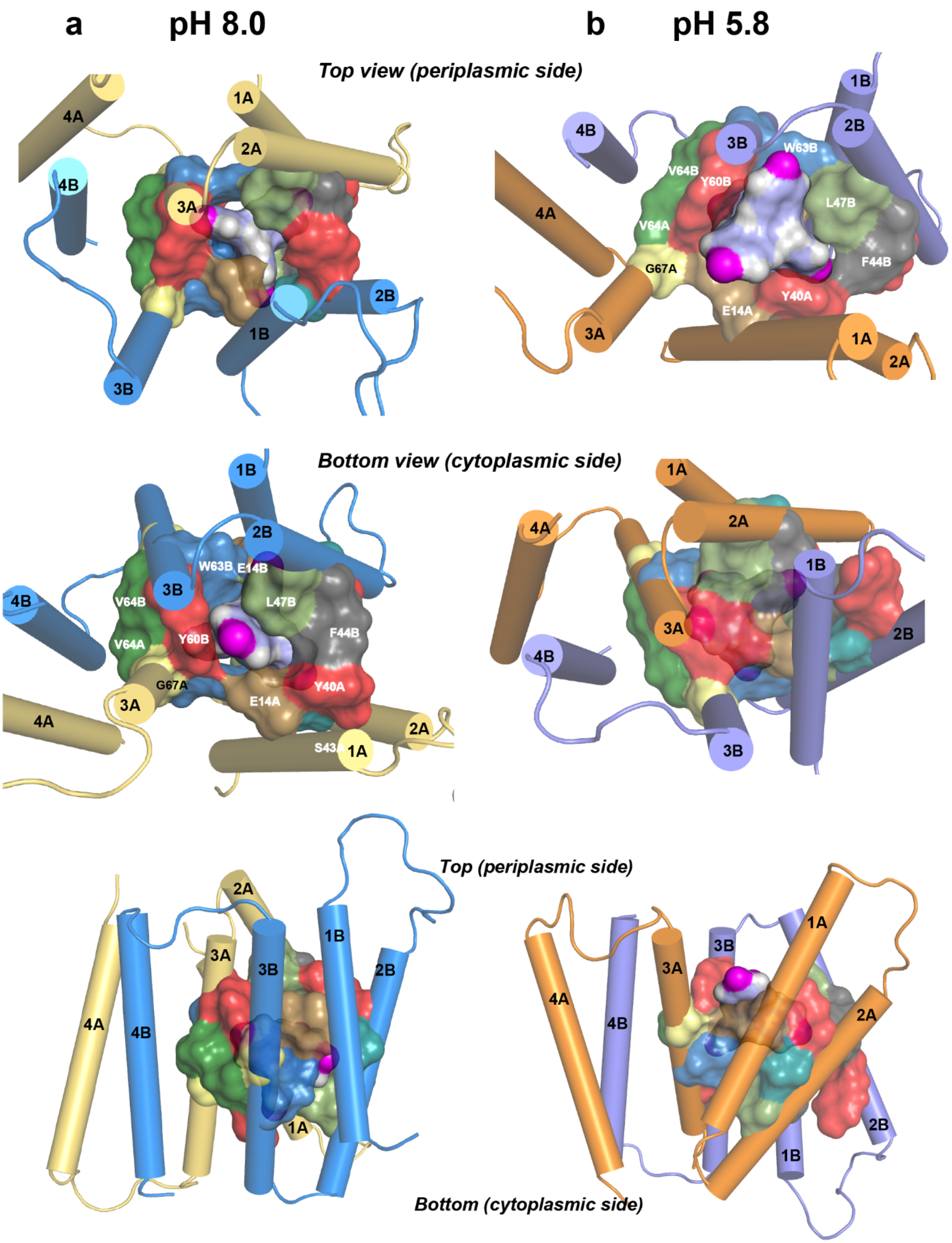

Supplementary Figure 7. Structural comparison of the EmrE-TPP complexes at high and low pH. (**a**) Surface views of the binding-site residues at high pH. (**b**) Surface views of the binding-site residues at low pH. Top and bottom sides are functionally defined (see main text). At high pH, F_4-TPP^+ is similarly and modestly exposed to the two sides of the helical bundle due to the relatively parallel orientations of the TM helices. In comparison, at low pH, F_4-TPP^+ is well exposed to the top of the helical bundle in a shallow binding pocket but is occluded at the bottom. We attribute the slower reorientation of the ligand at low pH to the bottom constriction in the low-pH complex.

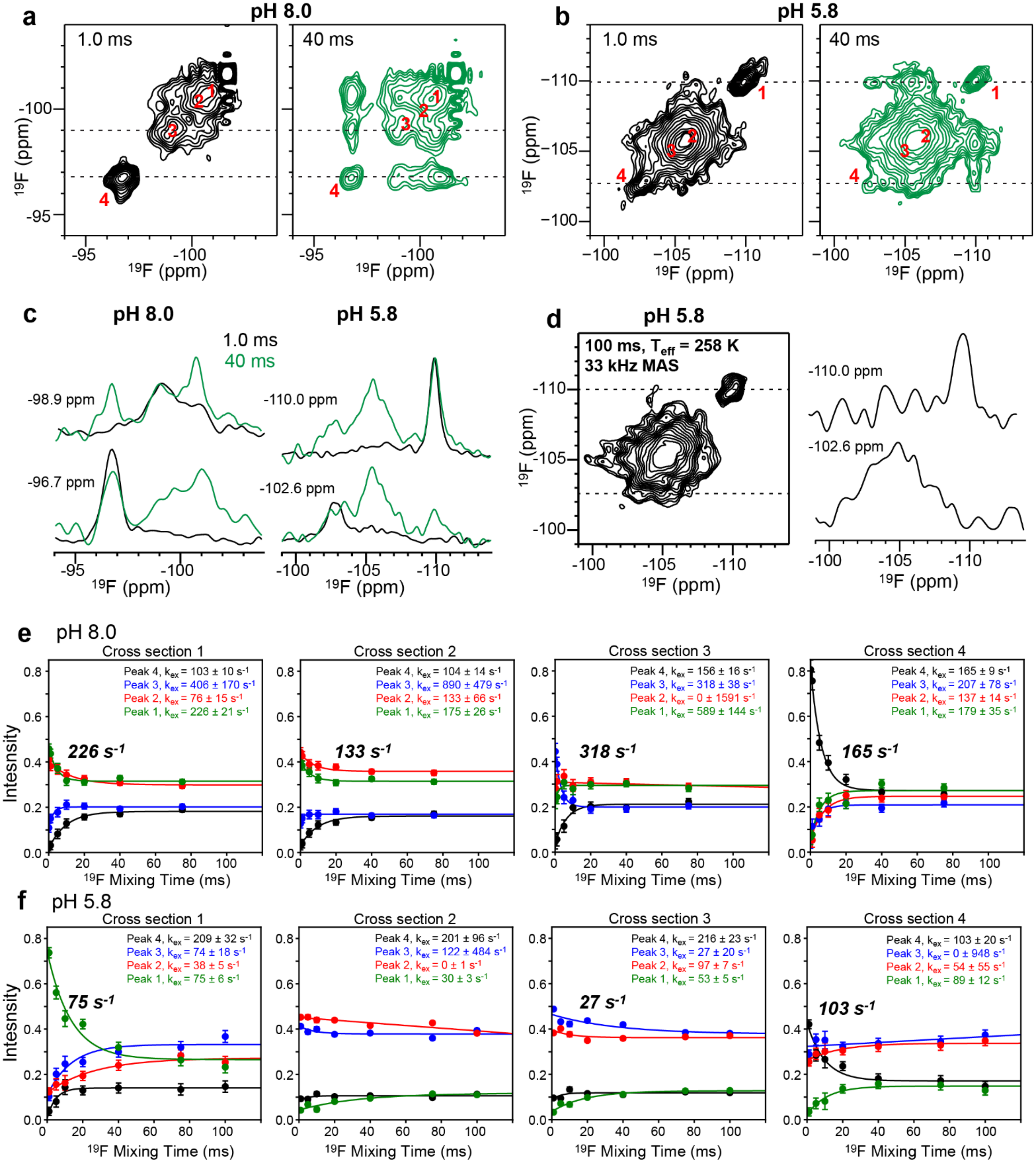

Supplementary Figure 8. Additional 2D ^{19}F-^{19}F exchange buildup curves of F$_4$-TPP$^+$ at high and low pH. (**a, b**) Representative 2D ^{19}F correlation spectra of F$_4$-TPP$^+$ with 1 ms and 40 ms mixing ms. (**a**) pH 8.0 data. (**b**) pH 5.8 data. These 2D spectra were measured at a sample temperature of ~285 K under 38 kHz MAS. (**c**) Two cross sections from the 2D spectra at both pH, showing the signal-to-noise ratios. (**d**) 100 ms 2D ^{19}F-^{19}F exchange spectrum of the pH 5.8 complex at an effective sample temperature of 258 K. No exchange cross peaks are observed, indicating that when immobilized, the inter-fluorine distances within each molecule are too long to be measured by spin diffusion on this timescale. Thus, the high-temperature cross peaks result from substrate reorientation. (**e**) Exchange buildup curves for the pH 8.0 sample. The diagonal peak's decay rates are indicated in each panel. (**f**) Exchange buildup curves for the pH 5.8

sample. The decay rates are smaller compared to the high-pH sample, indicating that the substrate reorients more slowly at acidic pH. Data are presented as mean values +/- 2σ. Error of intensity values was propagated from spectral signal to noise, while fitting parameter errors were estimated by Monte Carlo methods. The exchange peak intensities are provided as a Source Data file.

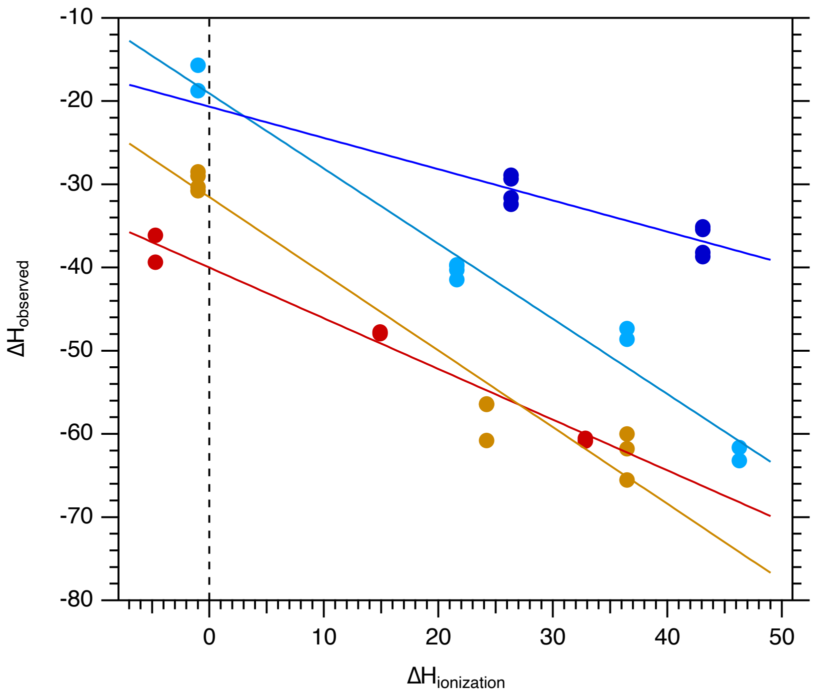

Supplementary Figure 9. Determination of pH-dependent binding enthalpy. Drug binding to EmrE releases protons, which are then bound by buffer, leading to a buffer-dependent contribution to the observed binding enthalpy. ITC experiments were performed for TPP^+ binding to WT EmrE in isotropic bicelles at 45°C at pH 5.5 (dark red), pH 6.5 (dark orange), pH 7.5 (cyan) and pH 8.5 (dark blue) with multiple buffers, as previously reported in (18). Extrapolation of the observed binding enthalpy to $\Delta H_{ionization}$ = 0 (dotted vertical line) allows determination of ΔH_{bind} due solely to the interaction of TPP^+ with the transporter, independent of the secondary protonation and deprotonation of the buffer. The thermodynamic data are provided as a Source Data file.

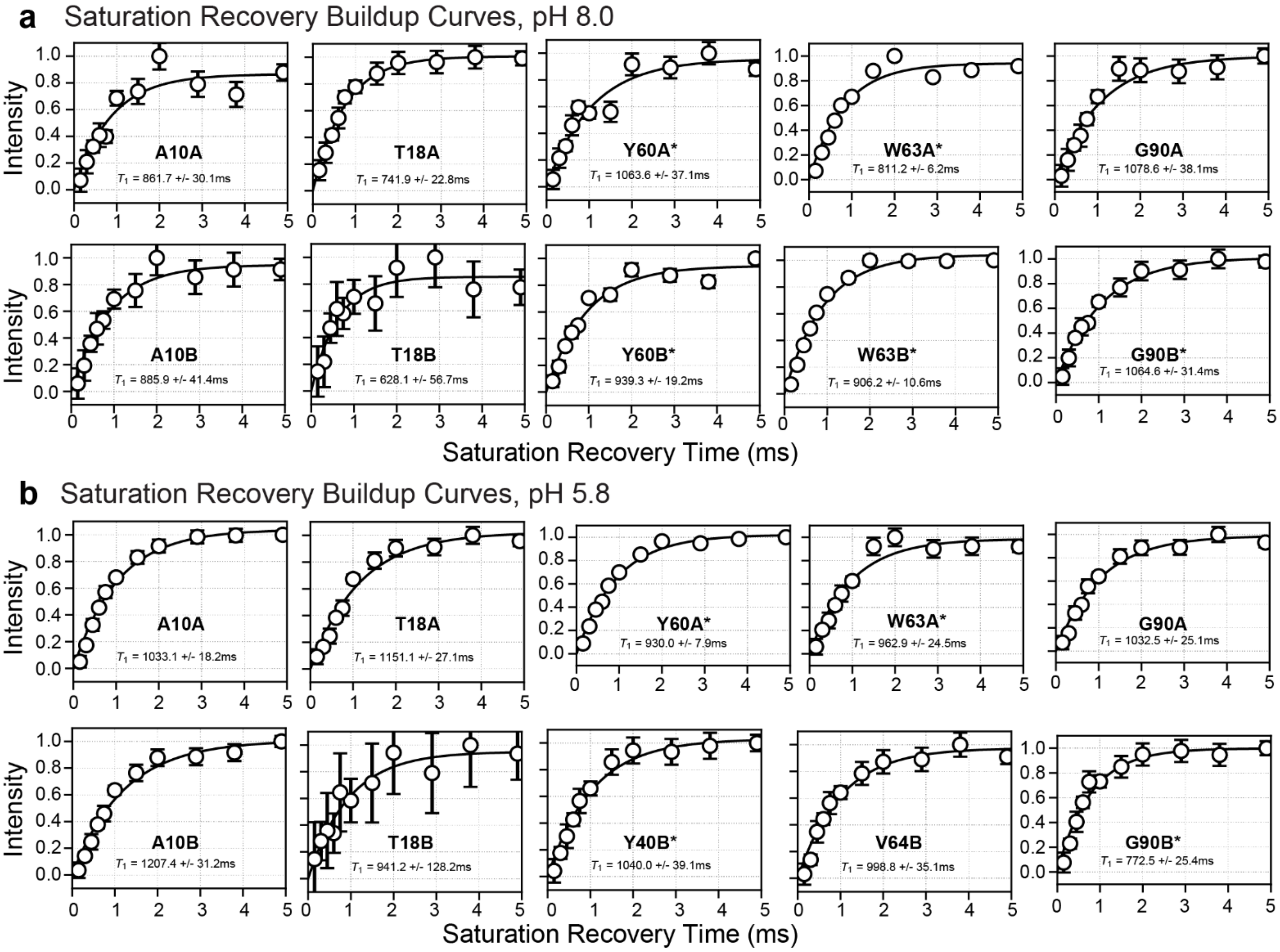

Supplementary Figure 10. Representative saturation recovery data of the EmrE-TPP complex. (**a**) High pH data. (**b**) Low pH data. Asterisks indicate residues that are partially overlapped in the 2D hNH spectra. Peak volumes were normalized by the largest value for each residue in the mixing time series and fit to a single-exponential plateau buildup in a two-parameter fit. Error bars were propagated from the signal-to-noise ratios (SNR) of each peak. Uncertainty of T_1 fitting parameter was determined by a Monte Carlo error estimation of 1000 trials and represents random uncertainty from the spectral SNRs and does not include systematic errors that result from peak shifts or spectral overlap. Data are presented as mean values +/- 2σ. Error of intensity values was propagated from spectral signal-to-noise ratios, while fitting parameter errors were estimated by Monte Carlo methods. Intensity values are provided as a Source Data file.

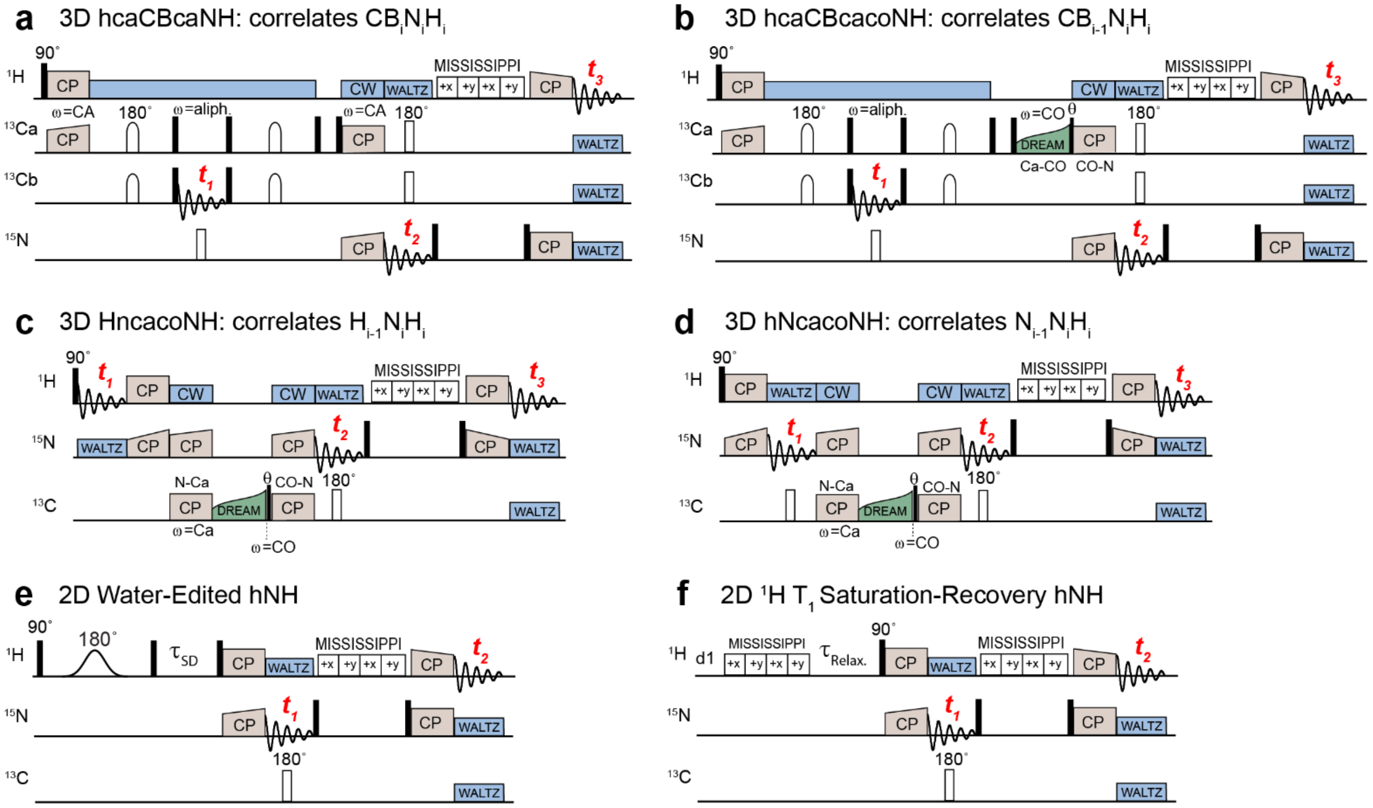

Supplementary Figure 11. Pulse diagrams for some of the 3D ^{1}H-detected correlation experiments for resonance assignment of EmrE at pH 8.0. (**a**) Intra-residue hcaCBcaNH experiment. (**b**) Inter-residue hcaCBcacoNH experiment. (**c**) Inter-residue HncacoNH experiment. (**d**) Inter-residue hNcacoNH experiment. (**e**) 2D hNH-resolved water-edited experiment. A selective 180˚ pulse is used to select the water magnetization, which is then allowed to diffuse to the protein amide protons during the variable delay τ_{SD}. (**f**) 2D hNH-resolved ^{1}H T_1 relaxation experiment. MISSISSIPPI solvent suppression is used prior to ^{1}H excitation to saturate the system, after which a variable delay τ_{relax} allows the system to undergo T_1 relaxation.
