# Supplementary material for: High-pH structure of EmrE reveals the mechanism of proton-coupled substrate transport
Source: Nat Commun. 2022 Feb 18;13:991. doi: 10.1038/s41467-022-28556-6 (PMC8857205; doi:10.1038/s41467-022-28556-6)
Supplement: Supplementary file 3 — Reporting Summary [file 41467_2022_28556_MOESM3_ESM.pdf]

nature portfolio

Corresponding author(s): Mei Hong

Last updated by author(s): Oct 24, 2021

Reporting Summary

Nature Portfolio wishes to improve the reproducibility of the work that we publish. This form provides structure for consistency and transparency in reporting. For further information on Nature Portfolio policies, see our Editorial Policies and the Editorial Policy Checklist.

Statistics

For all statistical analyses, confirm that the following items are present in the figure legend, table legend, main text, or Methods section.

n/a	Confirmed	
☐	☒	The exact sample size (*n*) for each experimental group/condition, given as a discrete number and unit of measurement
☐	☒	A statement on whether measurements were taken from distinct samples or whether the same sample was measured repeatedly
☒	☐	The statistical test(s) used AND whether they are one- or two-sided<br>*Only common tests should be described solely by name; describe more complex techniques in the Methods section.*
☒	☐	A description of all covariates tested
☒	☐	A description of any assumptions or corrections, such as tests of normality and adjustment for multiple comparisons
☐	☒	A full description of the statistical parameters including central tendency (e.g. means) or other basic estimates (e.g. regression coefficient) AND variation (e.g. standard deviation) or associated estimates of uncertainty (e.g. confidence intervals)
☒	☐	For null hypothesis testing, the test statistic (e.g. *F*, *t*, *r*) with confidence intervals, effect sizes, degrees of freedom and *P* value noted<br>*Give P values as exact values whenever suitable.*
☒	☐	For Bayesian analysis, information on the choice of priors and Markov chain Monte Carlo settings
☒	☐	For hierarchical and complex designs, identification of the appropriate level for tests and full reporting of outcomes
☒	☐	Estimates of effect sizes (e.g. Cohen's *d*, Pearson's *r*), indicating how they were calculated

Our web collection on statistics for biologists contains articles on many of the points above.

Software and code

Policy information about availability of computer code

Data collection	NMR data acquisition: Topspin 3.2, 3.6
Data analysis	NMR data processing and plotting: Topspin 2.1, 3.2 , 3.6<br>Peak picking and resonance assignment: Sparky 3<br>Torsion angle predictions: TALOS-N web server<br>1H-19F REDOR simulations: SIMPSON 4.1<br>Converting experimental restraints to GROMACS input files: Python 3.6<br>NumPy v1.20.3<br>SciPy v1.7.1<br>Pandas v1.3.4<br>MatPlotLib v3.4.3<br>MDAnalysis v1.0.0<br>Lmfit v1.0.1<br>Docking: HADDOCK 2.4<br>MD preparation: CHARMM-GUI 3.1<br>Structure calculation: GROMACS 2019.1

For manuscripts utilizing custom algorithms or software that are central to the research but not yet described in published literature, software must be made available to editors and reviewers. We strongly encourage code deposition in a community repository (e.g. GitHub). See the Nature Portfolio guidelines for submitting code & software for further information.

Data

Policy information about availability of data

All manuscripts must include a data availability statement. This statement should provide the following information, where applicable:

- Accession codes, unique identifiers, or web links for publicly available datasets
- A description of any restrictions on data availability
- For clinical datasets or third party data, please ensure that the statement adheres to our policy

Solid-state NMR chemical shifts and distance restraints have been deposited in the Biological Magnetic Resonance Bank (BMRB) with ID number 30957. The structural coordinates have been deposited in the Protein Data Bank with accession code 7SFQ.

Field-specific reporting

Please select the one below that is the best fit for your research. If you are not sure, read the appropriate sections before making your selection.

☒ Life sciences ☐ Behavioural & social sciences ☐ Ecological, evolutionary & environmental sciences

For a reference copy of the document with all sections, see nature.com/documents/nr-reporting-summary-flat.pdf

Life sciences study design

All studies must disclose on these points even when the disclosure is negative.

Sample size	NMR samples contained >10^16 molecules; acquired spectra report on the average over all molecules in the sample. A large number of scans were obtained for each NMR spectrum to generate signal-to-noise ratios of at least 5 to 1. Sample size was choosen by maximizing the amount of material that could be fit into the SSNMR rotor.
Data exclusions	No data was excluded.
Replication	Recombinant expression/purification and NMR spectra were highly replicable. NMR spectra were replicated hundreds of times for sample fingerprint checks over the course of 1 year. No chemical shift or sensitivity changes were observed in fingerprint spectra.
Randomization	Randomization was not relevant for this study, as samples consist of >10^16 randomly distributed molecules and signal averaging is the physical equivalent to randomization.
Blinding	Blinding was not relevant for this study, as no human subjects were studied. Physical data were processed exactly as described and no data were omitted from analysis; physical data is not biased by researcher input.

Reporting for specific materials, systems and methods

We require information from authors about some types of materials, experimental systems and methods used in many studies. Here, indicate whether each material, system or method listed is relevant to your study. If you are not sure if a list item applies to your research, read the appropriate section before selecting a response.

Materials & experimental systems

n/a	Involved in the study	
☒	☐	Antibodies
☒	☐	Eukaryotic cell lines
☒	☐	Palaeontology and archaeology
☒	☐	Animals and other organisms
☒	☐	Human research participants
☒	☐	Clinical data
☒	☐	Dual use research of concern

Methods

n/a	Involved in the study	
☒	☐	ChIP-seq
☒	☐	Flow cytometry
☒	☐	MRI-based neuroimaging
